# Senescent Phenotype of Astrocytes Leads to Activation of BV2 Microglia and N2a Neuronal Cells Death

**DOI:** 10.3390/molecules27185925

**Published:** 2022-09-12

**Authors:** Wenyou Zhang, Xuehan Yang, Jingyue Liu, Yichen Pan, Ming Zhang, Li Chen

**Affiliations:** 1 Nanomedicine Engineering Laboratory of Jilin Province, Department of Pharmacology, College of Basic Medical Sciences, Jilin University, Changchun 130021, China; 2 School of Life Sciences, Jilin University, Changchun 130021, China; 3 School of Nursing, Jilin University, Changchun 130021, China

**Keywords:** aging, microglia activation, senescent phenotype of astrocytes, cell-to-cell interaction, neurons

## Abstract

(1) Background: Astrocytes, the most abundant cell type in the central nervous system, are essential to tune individual-to-network neuronal activity. Senescence in astrocytes has been discovered as a crucial contributor to several age-related neurological diseases. Here, we aim to observe if astrocytes demonstrate senescence in the process of brain aging, and whether they bring adverse factors, especially harm to neuronal cells. (2) Methods: In vivo, mice were housed for four, 18, and 26 months. An in vitro cell model of aged astrocytes was constructed by serial passaging until passage 20–25, and those within 1–5 were invoked as young astrocytes. Meanwhile, an oxidative induced astrocyte senescence model was constructed by H_2_O_2_ induction. (3) Results: In vitro aged astrocytes all showed manifest changes in several established markers of cellular senescence, e.g., P53, P21, and the release of inflammatory cytokine IL-6 and SA-β-gal positive cells. Results also showed mitochondrial dysfunction in the oxidative stress-induced astrocyte senescence model and treatment of berberine could ameliorate these alterations. Two types of senescent astrocytes’ conditioned medium could impact on neuron apoptosis in direct or indirect ways. (4) Conclusions: Senescent astrocyte might affect neurons directly or indirectly acting on the regulation of normal and pathological brain aging.

## 1. Introduction

Aging is a major risk factor of numerous human diseases. As the elderly population has been expanding rapidly, the growing prevalence of age-related diseases is a major public concern. Arguably, one of the most devastating is the changes that occur in the central nervous system (CNS), leading to the loss of cognitive, motor, and emotional function that in essence make us who we are. During aging, all those brain cells could be undergoing the process of senescence. Recent studies have demonstrated that the clearance of senescent cells results in prolonged life span in mice [1]. Studies [2,3] published in Nature simultaneously reported that aging of organ is an “Asynchronous” process, and various types of cells as the basic unit of organ composition also follow this “Asynchronous” feature. The mammalian brain is composed of a multitude of cell types, e.g., astrocytes, microglia, and neurons, leaving the question: do precedent unfavorable factors occur among this different type of cells and how do they interact and affect each other?

Astrocytes are the predominant glial cells in the CNS and serve multiple functions including maintaining the formation of brain, secreting various extracellular matrix proteins and neurotrophic factors, also regulating the transmission efficiency at pre- and post-synaptic sites, and modulating synapse formation and turnover [2,4]. Thus, astrocytes play crucial roles in maintaining normal brain function and homeostasis [5,6]. Meanwhile, microglia, the resident macrophages of the CNS, have been widely considered as a homogeneous population of cells involved in stable brain patterns. Researchers have found that the activation of microglia has a great effect on cells in the brain, and the most common behavior of these types of cells is to release a large number of inflammatory factors, such as IL-1β, IL-6, and TNF-α, and finally affect other cells in brain. However, whatever the mechanism of astrocytes, microglia, other glia cells, or endothelial cells in brain, such a process will eventually lead to the functional loss or death of neurons, and ultimately manifest through a variety of physical behaviors. Questions arise as to how astrocytes, microglia, and neurons cross talk with each other.

In the present study, we first investigated whether neuronal damage occurs at the same time as aging and whether cognitive dysfunction happens at the whole animal level. Then, we explored primary cultures of rat astrocytes with natural senescence and oxidative stress-induced senescence to detect their interaction with microglia and neurons, and we found that berberine treatment may be an effective therapeutic approach to protect the mitochondria of astrocytes from malfunction.

## 2. Results

### 2.1. Cognitive Impairment and Neuron Loss Occurred in Aged Mice

**Figure 1:**
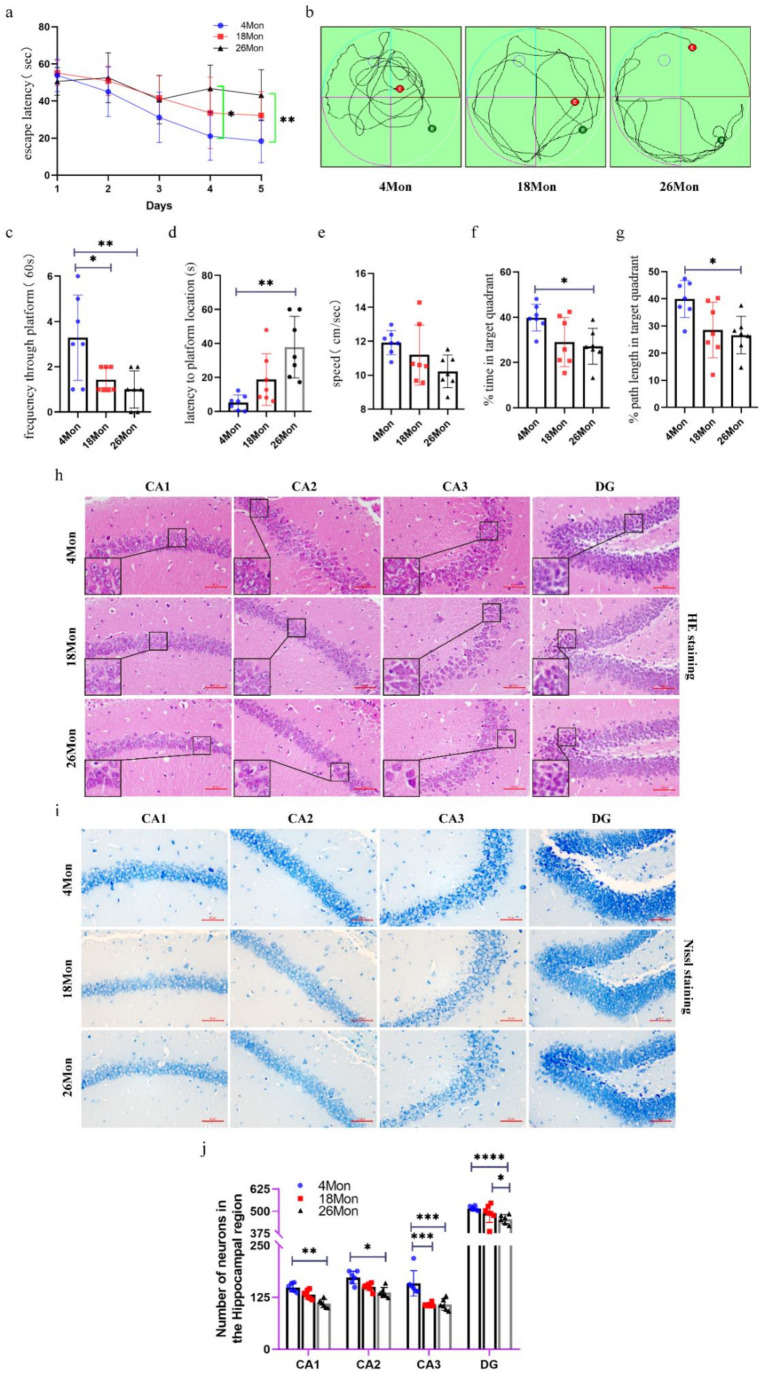
Age related cognitive impairment and neuron loss in mice. (**a**) Escape latency. (**b**) Movement Trajectory of mice. (**c**) Frequency through platform. (**d**) Latency to platform location. (**e**) The average swimming speed. (**f**) The percentage of time in target quadrant. (**g**) The percentage of path length in target quadrant. (**h**) HE staining of mouse brain. Scale bar, 50 μm. (**i**) Nissl staining in the hippocampus of each group. Scale bar, 50 μm. (**j**) The number of living neurons of hippocampus in mice (*n* = 6 mice/group). All experiments were expressed as the mean ± S.D, analyzed by ANOVA followed by Tukey’s test, * *p* < 0.05, ** *p* < 0.01, *** *p* < 0.001, **** *p* < 0.0001.

The Morris water maze test is a major behavioral testing method for measuring cognitive impairment of rodents. To further explore whether the changes in cognitive function associated with age, four-, 18-, 26-month-old male mice were chosen as our research subjects. As the training progressed, the latency to reach the platform was significantly decreased in the 26-month-old group at the fourth and fifth day (Figure 1a), indicating that the older mice showed significant learning deficiency during the sessions. Three typical movement trajectories on the sixth day are presented (Figure 1b), the time and path length in the target quadrant, the swimming speed, the tracking pathway and the latency to location were also analyzed (Figure 1c–g). The four-month-old mice spent the least amount of time to get the location of the original platform and took much more times swimming through the platform (Figure 1c,d). Compared with the four -month-old mice, the 18-month-old mice and the 26-month-old mice spent less time in the target quadrant along with the path length in the target quadrant (Figure 1f,g). In addition, the average speed was lower in the 18-month-old mice and the 26-month-old mice than in the four-month-old mice (Figure 1e), but without significant differences from each other. Since the data show differences in behavioral test, and the hippocampus is a crucial structure in memory circuits and is firstly and foremostly responsible for memory and learning functions, the HE [7] and Nissl staining were performed to see whether the structural integrity of the hippocampus was changed or the neurons start to lose. HE staining of the hippocampal neurons revealed that neurons in the group of four-month-old mice had rich cytoplasm, slightly round stained nucleus, and clear formation. On the contrary, in the group of 24-month-old mice, degenerated neurons showed shrinking cytoplasm and bodies, and most of the degenerated neurons revealed deep stained nucleus and irregular morphology (Figure 1h). The neuronal loss was evaluated with age. As shown in Figure 1i, four-month-old mice showed highly dense pyramidal layer neurons with intact structure. In contrast, the neurons appeared atrophied and pyknotic in 26-month-old mice. Meanwhile, the numbers were also lower than those in four-month-old mice, especially in the region of CA1, CA3, and DG, which can be seen intuitively from Figure 1j.

### 2.2. The Emergence of Senescent Astrocytes and Activated Microglia

**Figure 2:**
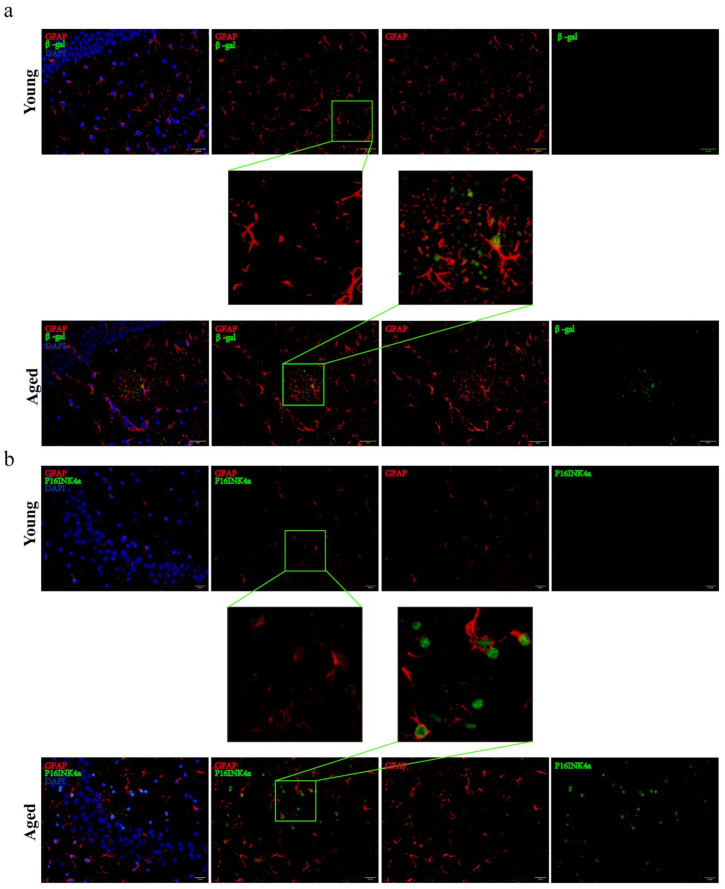

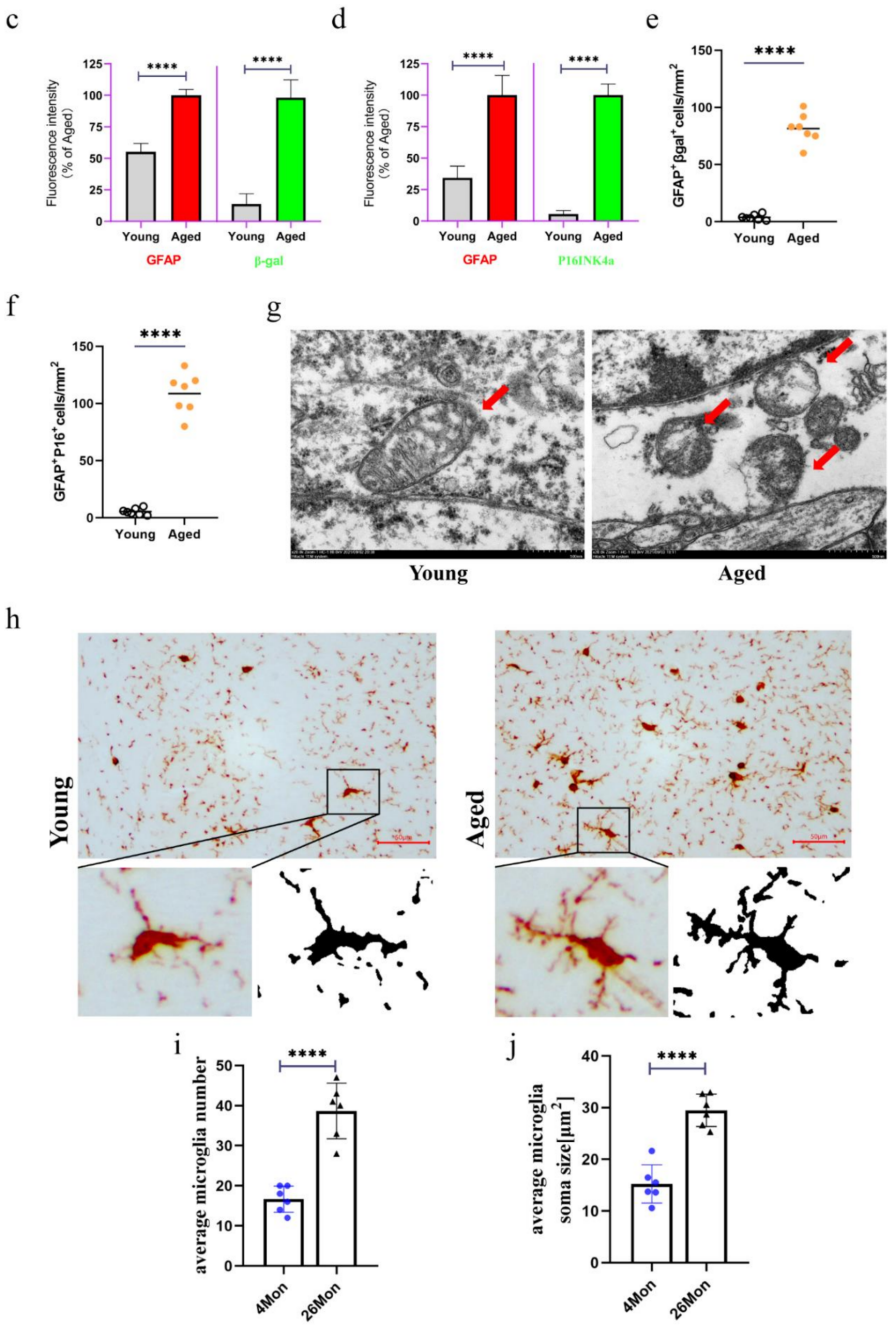
Senescent astrocytes occurred and accompanied with microglia activation. (**a**) Representative brain sections co-immunostained for GFAP (red) and β-gal (green) in young and aged mice (hippocampus). ×200 magnification. (**b**) Representative brain sections co-immunostained for GFAP (red) and P16^INK4a^ (green) in young and aged mice (hippocampus). ×200 magnification. ((**c**,**d**)) Quantification of data from (**a**,**b**) (*n* = 7 mice/group). (**e**,**f**) Analysis of the positive number of GFAP+βgal+ or GFAP+P16+ cells. (**g**)Transmission electron microphotographs of mitochondria in astrocytes from hippocampus of young and aged mice. Scale bar, 500 nm. (**h**) Brain sections immunostained for iba1. Scale bar, 50 μm. (**i**,**j**) Quantification of the average soma size and numbers of microglia in the CA3 hippocampal region (*n* = 6 mice/group) All experiments were expressed as the mean ±S.D, analyzed by ANOVA followed by Tukey’s test, ****p < 0.0001.

A variety of cells exist in the brain, mainly including neurons, astrocytes and microglia, which type of cells play a key role in the initiation of aging remains unclear. The expression of β-gal and P16^INK4a^ can be used as senescent markers [8]. Here, the four-month-old mice were defined as young group and the 26-month-old mice were defined as aged group. With the increasing age of mice, the expressions of β-gal and P16^INK4a^ were significantly increased (Figure 2a–d). We also found a significant increase in the density of βgal+ astrocyte (GFAP+), localizing in the cytoplasmic compartment and P16+ astrocyte (GFAP+), localizing in the nuclear compartment (Figure 2e,f). So, to assess whether changes in the organelle structure of astrocytes occur during the senescent progress, electron microscopy was used to examine the ultrastructure of the mitochondria in astrocytes. Figure 2g shows that the mitochondria may have changed from a strip shape to a rounded one with age and the cristae were vague and some of them disappeared in aged mice. Data also showed that a progressive enlargement of microglial size as the activation state and phagocytic capacity of the cells enhances (Figure 2h-j). Usually, amoeboid microglia was considered as higher activation state [9].

### 2.3. Serial Passaged Astrocytes Show Phenotypes of Aged Cells and Affected Neuronal Viability

**Figure 3:**
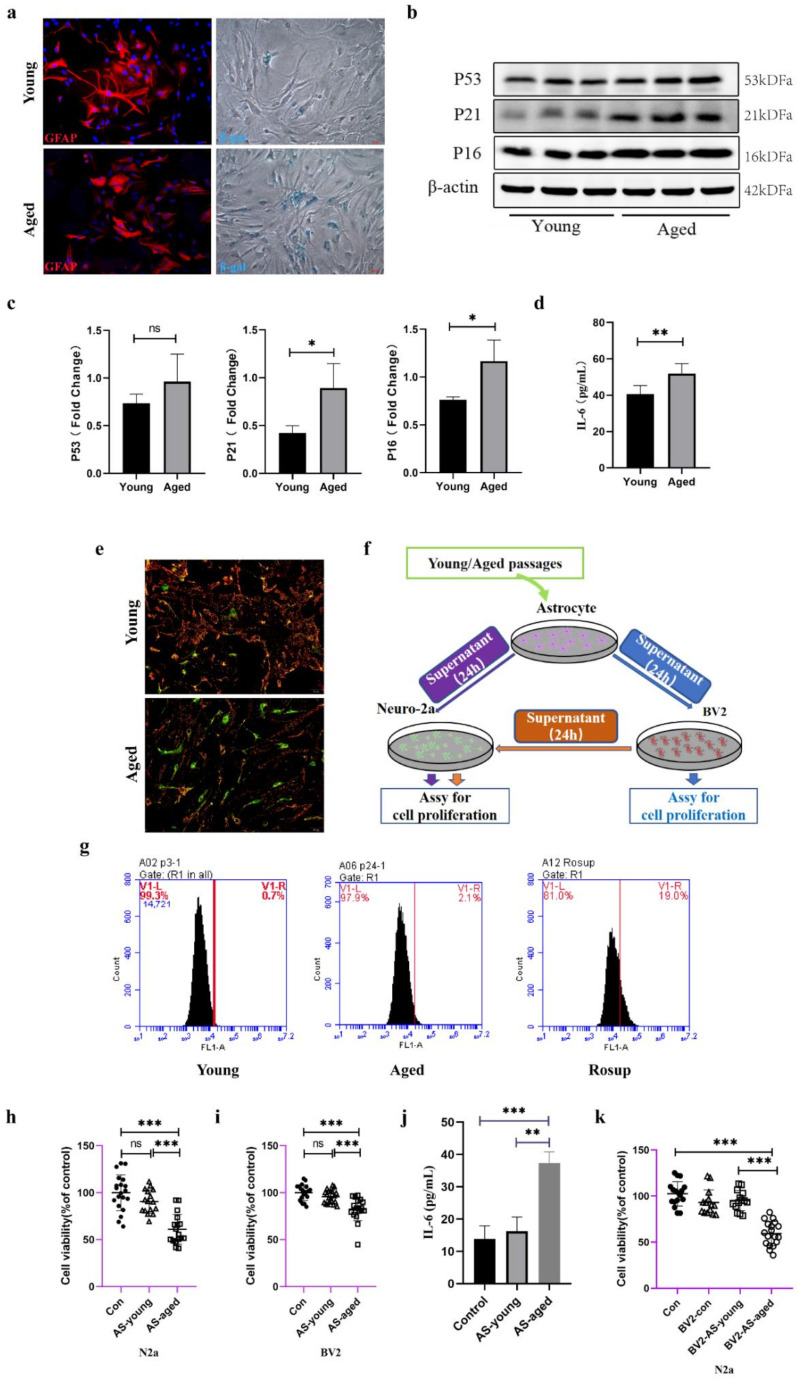
Astrocytes acquired senescent phenotype through serial passaging and SASP secretion may cause direct or indirect neuronal damage. (**a**) Immunofluorescent staining using GFAP antibody as astrocytes’ marker, and cell senescence staining of β-gal. (**b**) Representative immunoblots and (**c**) quantitation of P53, P21, P16 in young and aged astrocytes. Data are expressed relative to young, *n* = 3. (**d**) Cytokine ELISA of IL-6 in culture medium released from young and aged astrocyte, *n* = 4. (**e**) JC-1 staining. The red and green fluorescence reflects changes in the mitochondrial membrane potential of young and aged astrocytes. (**f**) Scheme of conditioned media (CM) and cell viability assays. (**g**) Intracellular ROS levels were measured using flow cytometry. (**h**) Cell viability assay of N2a cells treated with young and aged astrocytes’ supernatant, *n* = 20. (**i**) Cell viability assay of BV2 cells treated with young and aged astrocytes’ supernatant. (**j**) Cytokine ELISA of IL-6 in culture medium of BV2 treated with young/aged-astrocytes’ supernatant, *n* = 20. (**k**) Cell viability assay of N2a cells treated with supernatant of BV2 cells (treated with supernatant of young and aged astrocytes), *n* = 20. All experiments were expressed as the mean ± S.D, analyzed by ANOVA followed by Tukey’s test, *p < 0.05, **p < 0.01, ***p < 0.001, ns represents *p* > 0.05.

To deeply investigate the changes in a range of physiological functions of astrocytes during senescence in vitro, astrocytes were isolated from Wistar rat pups and made identification by the marker of GFAP (Figure 3a), the data showed highly purity of astrocytes. Natural senescent induction showed that SA-β-gal positive astrocytes were increased compared with those young astrocytes (Figure 3a). To systematically explore the altered phenotype of young and aged astrocytes, senescent markers of P53, P16, and P21 were tested, and, IL-6, which was collectively known as the senescence-associated secretory phenotype (SASP) [10] was also assessed. As is shown in Figure 3b,c, in aged astrocytes, the expression of P53, P21, and P16 was gradually increased, and the concentration of IL-6 in supernatant of aged astrocytes was elevated either (Figure 3d). Mitochondrial membrane potential was selected to detect whether the mitochondrial function remained normally, notably, the MMP showed significantly decreased in those aged astrocytes (Figure 3e). ROS production is the most common product upon cells facing oxidative stress, and compared with young astrocytes, the aged astrocytes showed increased intracellular ROS accumulation (Figure 3g). To assess whether these changes of astrocytes would have deleterious effects on microglia or neurons, we then examined the neurotoxicity of Neuro-2a cells treated with different conditioned medium (CM) (Figure 3f). The results showed that aged astrocytes derived CM or BV2 supernatants which were treated with those aged-astrocytes-derived CM significantly decreased Neuro-2a cell viability (Figure 3h,k), aged astrocytes-derived CM promoted a negative contribution to the survival of BV2 cells (Figure 3i), and the level of IL-6 in BV 2 supernatant was increased (Figure 3j), suggesting that substances released from aged-astrocytes may cause damage along with activating the BV2 cells and prompting cells to release inflammatory mediators to damage neurons.

### 2.4. Astrocytes Activate a Senescence Program in Response to Oxidative Stress and Showed Mitochondrial Dysfunction

**Figure 4:**
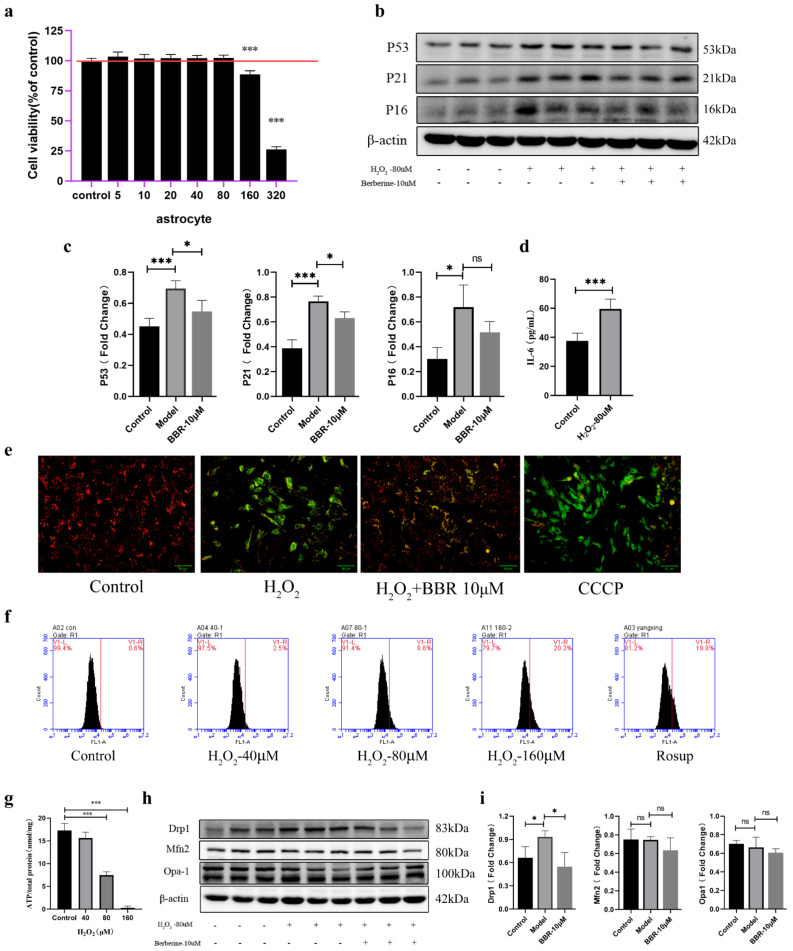
Mitochondrial functions in aged astrocytes were found declined and berberine may have relieved effect. (**a**) Cell viability assay of astrocytes treated with different concentration of H_2_O_2_, *n* = 8. (**b**) Representative immunoblots and (**c**) Quantitation of P53, P21, P16 and β-actin, in astrocytes treated with H_2_O_2_ and berberine. β-actin was a loading control and data are expressed relative to control, *n* = 3. (**d**) Cytokine ELISA of IL-6 in culture medium of astrocytes treated with or without H_2_O_2_, *n* = 4. (**e**) JC-1 staining. The red and green fluorescence reflects changes in the mitochondrial membrane potential of astrocytes treated with or without H_2_O_2_ and berberine, the group of CCCP was used as a positive control. *n* = 3. ×200 magnification. (**f**) Intracellular ROS levels were measured using flow cytometry. (**g**) ATP content was detected by the ATP Assess Kit, *n* = 4. (**h**) Representative immunoblots and (**i**) Quantitation of Drp1, Mfn2, Opa-1 and β-actin, in astrocytes treated with H_2_O_2_ and berberine. β-actin was a loading control and data are expressed relative to control, *n* = 3. All experiments were expressed as the mean ± S.D, analyzed by ANOVA followed by Tukey’s test, * *p* < 0.05, *** *p* < 0.001, ns represents *p* > 0.05.

Another in vitro mitochondrion damaged model of astrocytes induced with oxidative stress was conducted. During model construction, we found that the cell viability showed significant decrease when the concentration reached 160 μM (Figure 4a). In order to cause mitochondrial damage rather than apoptosis, 80 μM of H_2_O_2_ was used as modeling concentration. Next, senescence markers were also detected to find out if the cells reached the senescence phenotype. As the data show in Figure 4b,c, the proteins expression of P53, P21, and P16 was significantly elevated after treating with H_2_O_2_. The cell viability of astrocytes treated with different concentration of berberine showed significant decrease when the concentration reached 160 μM (Figure S1), and administration of berberine showed a certain degree of decrease in these indicators, although the expression of P16 decreased but showed no significance, and the concentration of IL-6 in supernatant of astrocytes treated with H_2_O_2_ was elevated compared with those that didn’t (Figure 4d). Mitochondrial membrane potential also showed that exogenous H_2_O_2_ treatment caused MMP decrease and administration of berberine would do recovery of the damaged MMP (Figure 4e). ATP production is the most common product produced by mitochondria, compared with control cells, H_2_O_2_ treatment markedly lowered cellular ATP levels (Figure 4g). The ROS level was also significantly increased after treatment of H_2_O_2_ (Figure 4f). What is more, H_2_O_2_ treatment might have an effect on mitochondrial fission by elevating the expression of mitochondrial fission protein drp1 and decreasing the expression of mitochondrial fusion proteins Mfn2 and opa-1, although the mitochondrial fusion protein showed no significant decrease. The treatment of berberine could have an ameliorating effect on the mitochondrial function caused by oxidative stress (Figure 4h,i).

### 2.5. Direct Interactions between Senescent Astrocytes and Neurons

**Figure 5:**
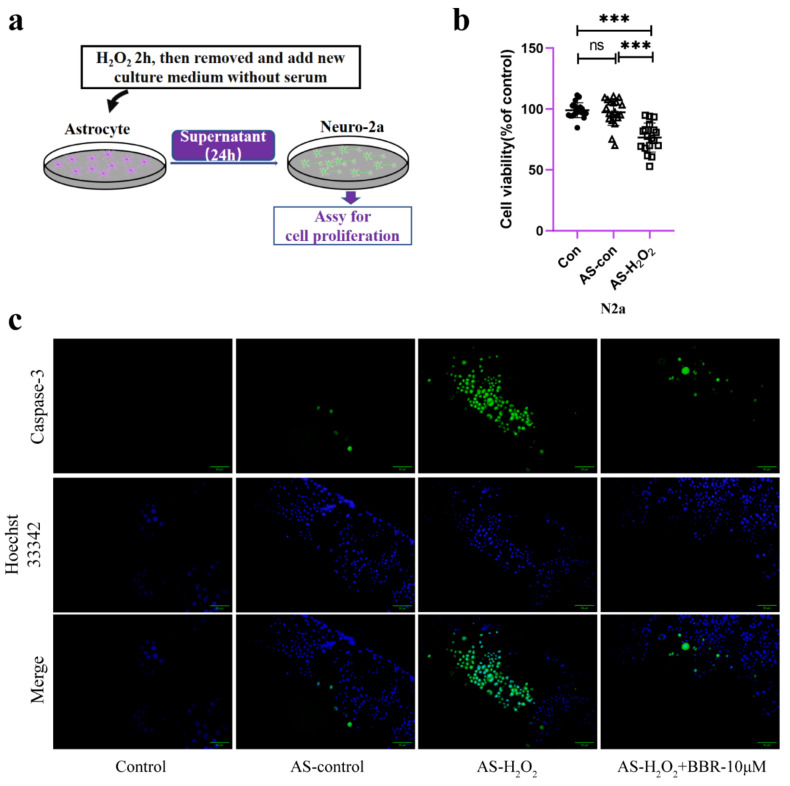
The direct effect of aged astrocytes in communicating with neurons. (**a**) A schematic diagram of the interaction of cell supernatants. (**b**) Cell viability assay of Neuro-2a cells treated with astrocyte-CM for 24 h, *n* = 20. (**c**) Caspase-3 activity in Neuro-2a cells (treated with astrocyte-CM for 24 h). Green fluorescence indicates the activity of caspase-3. Blue indicates nuclear stained by Hoechst33342. Merge is the overlapped green and blue. ×200 magnification, *n* = 4. All experiments were expressed as the mean ± S.D., analyzed by ANOVA followed by Tukey’s test, *** *p* < 0.001, ns represents *p* > 0.05.

To confirm that mitochondrial dysfunction of H_2_O_2_-treated astrocytes is responsible for neuronal viability, the neurotoxicity of Neuro-2a cells cultured with conditioned medium isolated from H_2_O_2_-stimulated astrocytes (Figure 5a) was examined. Data showed that H_2_O_2_-treated astrocyte-derived CM significantly decreased the viability of Neuro-2a cells (Figure 5b). However, CM from astrocytes treated without H_2_O_2_ did not affect the viability of Neuro-2a cells. To confirm that apoptosis happens after treating with conditioned medium for 24 h, we also detected caspase-3 activity of Neuro-2a cells and found that Neuro-2a cells treated with H_2_O_2_-treated astrocyte supernatant presented a significant increase of caspase-3 activity at 24 h (Figure 5c). What is more, this effect was ameliorated by the administration of berberine.

### 2.6. Indirect Effects of Senescent Astrocytes to Neurons

**Figure 6:**
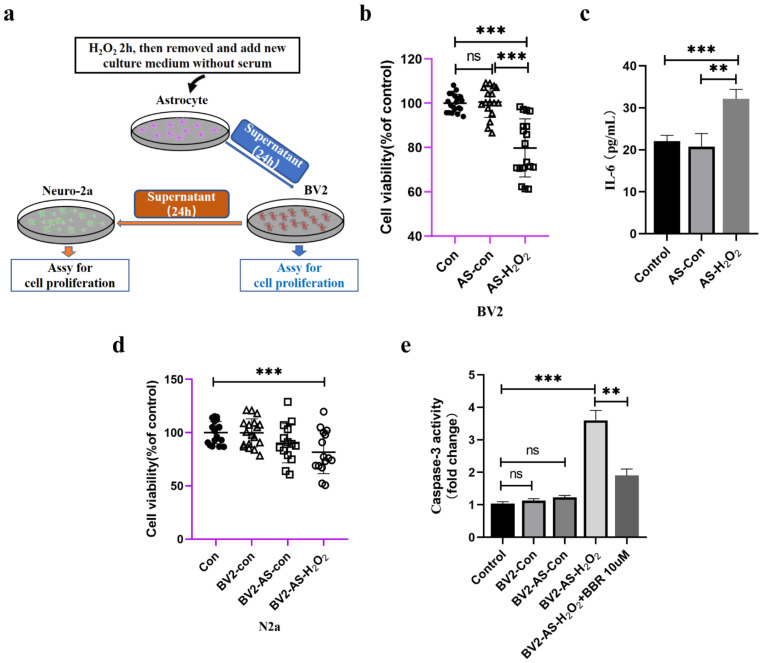
The indirect effect of aged astrocytes in communicating with microglia and neurons. (**a**) A schematic diagram of the interaction of cell supernatants. (**b**) Cell viability assay of BV2 cells by using astrocytes treated with or without H_2_O_2_ supernatant, *n* = 20. (**c**) Cytokine ELISA of IL-6 in culture medium of BV2 treated with astrocyte supernatant (treated with or without H_2_O_2_), *n* = 4. (**d**) Cell viability assay of N2a cells by using BV2 treated with astrocyte supernatant, *n* = 20. (**e**) The caspase-3 activity was measured with a caspase-3 assay kit, *n* = 6. All experiments were expressed as the mean ± S.D., analyzed by ANOVA followed by Tukey’s test, ** *p* < 0.01, *** *p* < 0.001, ns represents *p* > 0.05.

In this part, we checked if astrocytes could have indirect toxic effects on Neuro-2a cells by activating microglia cells, which act as immune cells in the brain, making them secrete neuronal inflammation cytokines, thus causing harm to neuronal cells. As in hypothetical diagram shown in Figure 6a, we first examined the viability of BV2 cells treated with supernatant of H_2_O_2_-stimulated astrocytes and found that after culturing with condition medium for 24 h, the viability of BV2 cells was significantly decreased (Figure 6b). IL-6 also seemed to be a neuronal toxic cytokine, and SASP was significantly elevated compared to those treated without H_2_O_2_ (Figure 6c). After treating with H_2_O_2_-treated astrocyte-derived CM for 24 h, the supernatant of BV2 cells was collected and cultured with Neuro-2a cells. Data showed that after incubation with the conditioned medium of BV2 cells, the viability of Neuro-2a cells decreased significantly compared to other groups (Figure 6d). Moreover, the activity of caspase-3 of these Neuro-2a cells showed that the supernatant of BV2 cells (treated with supernatant of astrocyte with or without H_2_O_2_ for 24 h) was also capable of increasing caspase-3 activity of Neuro-2a cells, and administration of berberine would have an ameliorating effects (Figure 6e).

## 3. Discussion

Astrocytes play curial physical and molecular roles in the mammalian brain. Any interference of their normal physiological function may lead to the pathology of central nervous system diseases. Hence, the aging of astrocytes may have an immense impact on the function and micro-environment of the brain. Several researches demonstrated that aging [11] and diseases [12] cause loss of normal functions in astrocytes, which reduces their ability to properly maintain a healthy CNS environment. As a potential candidate of aging, cellular senescence was regarded as the inducing factor of aged-related neurodegenerative diseases. However, knowledge of the impact of senescent astrocytes in the brain is fragmented, and what’s more troubling is that it remains unclear how these senescent astrocytes may interact with microglia or neurons or any other types of cells in the mammalian brain. In our in vivo aging model of mice, we found that aged mice required more time to learn how to reach the platform than those young mice. Moreover, the hippocampus of old mice showed thinning and loosening of pyramidal cells, as well as pyknotic and atrophied neurons, while the relative neuron loss of young mice was less than that of old mice. Additionally, the activation of microglia is more severe than those in young mice, which consistent with findings in other studies [13,14]. Through the co-immunostaining of β-gal/P16^INK4a^ and astrocyte marker GFAP, it could be found that this cell senescent marker appears in astrocytes, and transmission electron microphotographs show that the mitochondria in aged mice may undergo malfunctioning, which suggests that astrocytes senescence may have occurred in the process of brain aging due to the mitochondrial dysfunction. Researchers have found that dysfunction of astrocytic mitochondria can cause deleterious actions on neurons [15,16] and may bring adverse factors to other types of cells in brain [17]. Therefore, we made attempts to establish astrocyte models of aging in vitro to assist in the study of brain aging.

Several reports have shown that late passage of primary or mesenchymal stem cells could appear the sign of senescence. Cultured primary MEF cells aged through later passage, which causes the cells to swell and eventually stop proliferating [18,19]. The replicative senescence of astrocytes has also been confirmed in primary cultures derived from normal or post-AD brain tissue [20,21] During our experiments on astrocytes and adipocyte-derived mesenchymal stem cells, it was found that higher passage number cells have indeed reduced proliferative and differentiation capabilities (data not shown). Therefore, we here constructed a replicated aging model and oxidative stress model of astrocytes to imitate senescent cells during aging. Actually, previous articles have reported astrocytes that underwent serial passage cultivation or oxidative stress showed aged phenotypes like nuclear enlargement [21,22], elevated expression of P53 and P21 and P16 [23]. Senescence-associated beta-galactosidase, which is overexpressed and accumulated in the lysosome specifically in senescent cells [24], was also widely used as a cellular senescence marker. In vitro models in our study also found these similar changes of indicators. What’s more is that the mitochondrial function of senescent astrocytes may decline with increased passaging [25], especially the level of proton leak and ATP, but we found that increased passaging did not affect the level of ATP (Figure S2), which is different from previous research, speculating that this discrepancy may be caused by the difference of length of culturing time and self-regulation of astrocyte itself, but the ATP level of the model of oxidative stress of astrocyte indeed changed. We also examined the mitochondrial membrane potential (MMP) and molecular changes in senescent astrocytes, finding that no matter the serial passing model or the oxidative model of senescent astrocytes model, MMP decline appeared, which suggests that mitochondrial function is negatively affected. However, indeed, a drop in mitochondrial membrane potential does not necessarily lead to the loss of mitochondrial functions, as research has reported that in Huntington’s disease occurred imbalanced mitochondrial fusion-fission [26]. Our results showed that oxidative stress induced astrocyte senescence occurred with elevated expression of DRP1, suggesting that fission in mitochondria of this type of senescent astrocytes was enhanced. Administration of berberine could ameliorate these mitochondrial dysfunctions caused by H_2_O_2_. Thus, the question remains concerning how mitochondrial dysfunction of senescent astrocytes impact on the process of brain aging.

It has been proposed that astrocytes can establish communications with microglia, neurons and other astrocytes [27], and may involve in synapse formation and elimination to different degrees in a wide range of disease conditions [28]. Astrocyte-microglia are deemed to have immune-related functions in the mammalian brain. Initially, all the inflammatory responses or regulatory process are activated with the disruption of steady-state brain homeostasis. This rapid response induces the process of brain repair mostly by those activated glia cells. However, sustained secretion of inflammatory mediators of astrocyte or microglia may induce chronic inflammation, which usually happens in brain aging and finally may contribute to neurodegeneration and cognitive decline [29,30]. Recently, research has found that a subset of microglia, named disease-associated microglia, are associated with the genes found in the human genome that is linked to Alzheimer’s disease (AD) and other neurodegenerative conditions [31,32,33]. This type of microglia will sustained secrete IL-6 and impact on neurons and finally lead to neuroinflammation. However, regarding how the microglia be activated, numerous explanations are aroused. Some research suggests that microglial may be activated by a repertoire of pattern recognition receptors (PRRs) which allow microglia to detect “harmful signals”, such as substances containing pathogen-associated molecular patterns (PAMPs) and damage-associated molecular patterns (DAMPs), including ATP or DNA, or different types of interleukins released from astrocyte or other types of cells in brain [34,35]. Upon detecting these messages, microglia migrate to those damaged positions and engulf those materials with an amoeboid-like “reactive” morphology [36,37]. Our study found that in the brain of aged mice, the microglial morphology was significantly altered, and in vitro data also found that serial passaging or oxidative stress induced senescent astrocytes secreted higher IL-6 compared to those young-astrocytes, so these mitochondrial dysfunction or interleukin release by astrocyte may be a cause of microglia activation and finally lead to neurons death due to the sustained secrete of neuroinflammatory cytokines. Our cell viability assay of astrocyte-microglia-neuron also proved this cell-to-cell interaction would eventually lead to neuronal death.

Except for the astrocyte-microglia-neuron interaction referred above, our data also suggest that different types of senescent astrocytes could do damage to neurons directly. Although astrocytes were initially considered as nonfunctional fillers of the neuronal network. However, with time and technology advances, the significance of this type of cell for many complicated biological processes has been elucidated. In vivo, they interact closer with neurons and participate in the “tri-partite synapse”, which couples neurotransmission between pre- and postsynaptic materials [38]. Further contributions include, e.g., against trauma, infection, and neurodegeneration to maintain neuronal health. However, many studies have reported that astrocyte senescence may directly influence neuronal health through multiple processes, e.g., SASP, P16, and P21 [23,39], which were referred to above, and DNA damage induced by ionizing radiation [40]. What’s more, research also reported that ROS-induced senescence in human astrocytes showed downregulated genes of neuronal development and differentiation and upregulated genes of proinflammation [41]. These may be partially responsible for the neuronal damage or death. In our study, we found that after neurons were incubated with the supernatant of senescent astrocytes, the viability was dramatically decreased, combined with elevated caspase-3 expression compared to those astrocytes treated without H_2_O_2_. These adverse effects were ameliorated by pre-treating with berberine when inducing oxidative stress with H_2_O_2_. These results suggest that senescent astrocytes could do impact on neuronal survive in a direct way.

Meanwhile, we used berberine as a positive control to deal with the dysfunction of mitochondria induced by oxidative stress, since it had long been reported to have the ability to maintain the normal function of mitochondria and previous studies also proved that it could possess several pharmacological properties [42,43,44,45], including anti-inflammatory, antifibrotic, and correcting the fission of mitochondria. Our data show that berberine may have the favorable potency of astrocytes from escaping the process of senescence.

## 4. Materials and Methods

### 4.1. Animals

Male C57bl6/J mice (8-week-old) were purchased from Vital River Laboratory Animals Technology Co, Ltd. [SCXK(Jing)2016-0006], housed in a temperature and climate condition-controlled barrier system (23 ± 2 °C and 45~60% relative humidity, 12 h light-dark cycle) and fed regular rodent chow (Laboratory Animal Center of Jilin University). Animal welfare and experimental procedures complied with the Provisions and General Recommendations of the Chinese Experimental Animals Administration Legislation. All animal experimental procedures were approved by the Ethics Committee for the Use of Experimental Animals of College of Basic Medical Sciences, Jilin University (SCXK(Jing)2014-0004). The animals were divided into 3 groups for analysis: 4-month-old mice (young group; *n* = 10), 18-month-old mice (middle-age group; *n* = 10), 26-month-old mice (aged group; *n* = 10). All animal experimental procedures were approved by the Ethics Committee for the Use of Experimental Animals of College of Basic Medical Sciences, Jilin University (SCXK(Jing)2014-0004). Mice were sacrificed under isoflurane inhalation anesthesia until indicated months of age, and then the brains were removed.

### 4.2. Tissue Processing

Fresh brain tissues were dissected and soaked overnight in 4% paraformaldehyde, dehydrated in an ascending ethanol series, and equilibrated with xylene, followed by embedding in paraffin and sectioning into 6μm slices. Then, samples were dewaxed with xylene and a descending ethanol series.

### 4.3. Reagents

Berberine dilutions were obtained from a 10 mM berberine chloride stock solution prepared in Dimethyl sulfoxide.

### 4.4. The Morris Water Maze (MWM) Test

The Morris Water Maze (MWM) test includes two main parts: the place navigation test and the spatial probe test. The study was conducted in a quadrant of a 1.8-m-diameter pool with an 8.5-cm-diameter platform submerged in opaque water. Water remained at temperature (22° ± 1 °C) through all trails. Distinct visual cues were present in all trials. The place navigation test was performed by giving four trials a day with 20- to 30-min intervals between trials for 5 days. The spatial probe test took place 24 h after the last place navigation test. The spatial probe was performed on the sixth day with the escape platform removed, and the probe trials were conducted for 60 s. The training and probe trials were recorded by a video camera mounted on the ceiling, and data were analyzed by using SMART v.3.0.06 (a product of Panlab Harvard Apparatus^®^, RWD Company, Shenzhen, China).

### 4.5. Cell Culture and Primary Astrocytes Culture

Mouse BV2 and Neuro-2a(N2a) cell lines were purchased from the National Collection of Authenticated Cell Cultures (Shanghai, China) and were maintained in DMEM/F12 supplemented with 10% fetal bovine serum (FBS), 2 mM l-glutamine, 100 IU·mL^−1^ penicillin, 100 μg·mL^−1^ streptomycin and reseeded at a 1:7 dilution every 3 days. Primary rat astrocytes were isolated from 1–2-day-old Wistar rat pups as described previously [46]. In brief, cortices were removed from the rat pups; the meninges were stripped and homogenized. After incubation with trypsin (0.05%) for 30 min in a 37 °C thermostatic shaker, the homogenate was resuspended in a trypsin inhibitor/DNase solution, triturated, and dissociated cortical cells were suspended in DMEM/F12 (Life Technology, MA, USA) containing 25 mM glucose, 4 mM glutamine, 1 mM sodium pyruvate, 100 IU·mL^−1^ penicillin, 100 μg·mL^−1^ streptomycin, and 10% FBS and plated on poly-L-lysine coated 10 cm dishes at a density of 1 × 10^5^ cells cm^2^ at 37 °C with 5% CO_2_ in air. After 24 h, the culture medium was changed to fresh medium. Monolayers of type 1 astrocytes were obtained 7 days after plating. Non-astrocytic cells, such as microglia and neurons, were detached from the flasks by shaking and removed by changing the medium. Cells were trypsinised and reseeded at a 1:3 dilution every 3 days on poly-L-lysine coated 10 cm dishes.

### 4.6. Senescence Induction

Induction of astrocyte senescence by oxidative stress was performed as previously described [47]. In brief, early-passage (no more than five passages) astrocytes were incubated for 24 h, then the medium was removed and 80 μM H_2_O_2_ in complete astrocyte culture medium was added for 2 h. Then, the medium was removed and washed with PBS, before cells were cultured in medium without serum for 24 h. Induction of natural senescent astrocytes was performed by trypsinizing astrocytes. When cells reached 80–90% confluency, primary rat astrocytes isolated from 1–2-day-old Wistar rat pups were defined as passage 0. The number of passages increases when cells are trypsinized once. Cells within passage 20–25 were used as aged-astrocytes and within passage 1–5 were used as young-astrocytes.

### 4.7. Senescence-Associated-Β-Galactosidase (Sa-β-Gal) Staining

β-galactosidase activity was measured using the Senescence β-Galactosidase Staining Kit (Beyotime, Shanghai, China). Briefly, cells were washed with PBS and fixed with 1× fixative solution for 15 min. Then, β-galactosidase staining solution with a final pH between 5.9 and 6.1 was prepared and added to the fixed cells. Samples were sealed with parafilm to prevent evaporation and placed in a 37 °C incubator without CO_2_ overnight. Imaging was performed using an inverted microscope.

### 4.8. Preparation of Astrocyte-Conditioned Medium and Microglia-Conditioned Medium

Astrocytes were grown in 96-well plates, when reaching 80% confluency, cells were treated with 80 μM H_2_O_2_ or normal culture medium for 2 h, then the medium was removed and washed with PBS for 1 time and DMEM/F12 without serum was added for 24 h, finally the supernatant was collected for downstream experiments. Conditioned medium from microglia was collected after treating with astrocyte conditioned medium for 24 h.

### 4.9. MTT Assay

Cell viability was determined using a modified MTT assay [48] Briefly, 5000 cells per well were plated in 96-well plates and incubated overnight. Cells were then treated with conditioned medium for 24 h. At the end of the follow-up period, MTT was added, and the cultures were incubated for 2 h at 37 °C in an incubator. After discarding the supernatant, the formazan was dissolved in DMSO. Then, the optical density (OD) values were determined at 492 nm. The cell viability was calculated by taking the cell viability in the non-treatment group as 100%.

### 4.10. ATP Measurement

Intracellular ATP level was determined by ATP assay kit (Beyotime, Shanghai, China), which can perform cell lysis and generate a luminescent signal proportional to the amount of ATP present. The preparation of samples was conducted according to the manual of the product. The supernatants of each sample (20 μL) were added to the ATP detection solution (100 μL) attached to the kit. Then, Infinite 200 Pro (Tecan, Mannedorf, Swiss) was utilized to record the RLU values. The protraction of the standard curve was conducted on the basis of the RLU values of ATP with the concentration of 0, 0.01, 0.05, 0.1, 0.5, 1, 5, and 10 nmol/L. Finally, the protein concentration was used to standardize the results, which were presented as ATP/protein (nmol/mg).

### 4.11. Mitochondrial Membrane Potential

Mitochondrial membrane potential (ΔΨm) loss was assessed by a Mitochondrial Membrane Potential Assay Kit with JC-1. After treatment for 24 h, cells were incubated with JC-1 for 20 min, washed and visualized under BX53 fluorescence microscope (Olympus, Tokyo, Japan). Red fluorescence indicates normal ΔΨm with JC-1 aggregates in mitochondria, and green reflects cytosolic JC-1 monomer indicating ΔΨm loss.

### 4.12. Western Blot Analysis

The harvested cells were digested by RIPA, followed by sonication. Samples were centrifuged for 15 min at 12,000× *g* at 4 °C. BCA Protein Assay Kit (Thermo Fisher, MA, USA) was applied to determine the total protein concentration. After separating the proteins on SDS-PAGE, the proteins were transferred onto the PVDF membrane (Millipore, MA, USA). In all, 5% non-fat milk was utilized for the sealing of membranes in Tris-buffered saline (pH 7.5). The membranes were then incubated with the primary antibodies and second antibodies. The protein bands were revealed by an ECL kit (Thermo Fisher, MA, USA). The expression levels of proteins were evaluated by Image J (National Institutes of Health, MD, USA). The primary antibodies were: anti-P53 (Proteintech, Wuhan, China), anti-P21 (Proteintech, Wuhan, China), anti-P16 (Abcam, Cambridge, UK), anti-OPA-1 (Proteintech, Wuhan, China), anti-Mfn2 (Abcam Cambridge, UK), anti-DRP-1 (Proteintech, Wuhan, China), anti-β-actin (Proteintech, Wuhan, China).

### 4.13. HE Staining

Fixed, paraffin-embedded brain tissue was sectioned and underwent hematoxylin eosin (HE) staining according to the following procedure. Sections were deparaffinized, washed in distilled water, and incubated in hematoxylin solution for 5 min; excess hematoxylin solution was washed off with running tap water. To remove background staining, sections underwent a differentiation step in hydrochloric acid alcohol with fully washed in running tap water. Sections were then counterstained in eosin solution for 2–3 min, washed in running tap water, dehydrated through graded alcohol, and mounted with neutral resin. Pathological changes in neurons were observed under a light microscope CX31 (Olympus, Tokyo, Japan), brain sections were acquired with 100× magnification.

### 4.14. Nissl Staining

Paraffin-embedded, fixed brain tissue was deparaffinized, washed 1–2 min in distilled water, dipped in 1% thionin lysol at 37 °C for 30 min, and washed again for 1–2 min in distilled water. To moderately differentiate the nucleus, sections were incubated in 0.5% hydrochloric acid alcohol, washed back to blue, and differentiated using 95% alcohol until the Nissl substance was visualized. Sections were then dehydrated (twice for 5 min each) in 100% anhydrous alcohol, permeabilized with xylene twice (5 min each), and mounted with neutral gum. Changes observed in neurons and Nissl bodies of the hippocampal CA1, CA2, CA3, and DG regions were detected under a light microscope CX31 (Olympus), brain sections were acquired with 100× magnification. The number of neurons was measured and quantified using ImageJ software.

### 4.15. Immunofluorescence and Immunohistochemistry

Total cells on the slides went through permeabilization in 0.3% Triton X-100 after the fixation in 4% paraformaldehyde. After that, goat serum was utilized for blocking. Cells or tissues were later cultivated overnight with anti-GFAP (Abcam, Cambridge, UK). Then, the slide was subjected to 1-h incubation with the Fluorescein (PE)-conjugated Affinipure Goat Anti-Mouse IgG(H+L) (Proteintech, Wuhan, China) at room temperature. Again, cells or tissues were cultivated overnight with anti-beta-Galactosidase (Proteintech, Wuhan, China) or P16INK4a (Abcam, Cambridge, UK) at 4 °C and then subjected to 1 h incubation with the FITC-conjugated Goat Anti-Rabbit IgG(H+L) (Proteintech, Wuhan, China). The counterstaining of the nucleus was accomplished by using DAPI. For immunohistochemistry (IHC), slides were incubated in 0.9% H_2_O_2_ for 30 min. Afterwards, the slides were placed in blocking buffer (goat serum 1:20 in PBST/BSA) for 30 min at room temperature. Then, anti-iba1 (Abcam Cambridge, UK) antibody was used, and tissues were further blocked with Biotin for 10 min each. Antibodies were detected using a rabbit peroxidase ABC Kit (MXB Biotechnologies, Guangzhou, China). Each sample was viewed with BX53 Fluorescence microscope (Olympus, Tokyo, Japan).

### 4.16. Iba1^+^ Cell Density and Soma Size Quantifications

Scans of the hippocampus were imaged using BX53 Fluorescence microscope (Olympus, Tokyo, Japan) in bright field on IHC sections stained for Iba1. The number of Iba1+ cells and the area were recorded. Cell numbers were expressed as number of Iba1+ cells per mm [49] in a 6 µm thick section. The area of the soma of Iba1+ cells was manually traced and measured in ImageJ.

### 4.17. Elisa Assays

Rat IL-6 Elisa kit (Thermo Fisher, MA, USA), Mouse IL-1 beta Elisa kit (Dakewe Biotech, Shenzhen, China), Rat IL-1 beta Elisa kit (Proteintech, Wuhan, China), Mouse IL-6 Elisa kit (Proteintech, Wuhan, China). Young/aged astrocytes were plated on 12-well plates. After reaching confluency, the medium was removed and DMEM/F12 without serum was added for 24 h. Rat IL-6 and IL-1 beta were detected by collecting the supernatant. Similarly, after culturing with H_2_O_2_ treated astrocytes conditioned medium for 24 h, the supernatant of BV2 was collected for detecting Mouse IL-6 and Mouse IL-1 beta.

### 4.18. Caspase-3 Activity Determination

Caspase 3 Activity Assay Kit and GreenNuc™ Caspase-3 Assay Kit for Live Cells was purchased from Beyotime (Nanjing, China) and was used to calculate caspase-3 activity in cells according to the manufacturer’s introductions.

### 4.19. Transmission Electron Microscopy

Fresh Brain tissue was cut into 1 × 1 × 2 mm^3^ samples, fixed in 2.5% glutaraldehyde solution, embedded, and sliced into ultrathin sections. The ultrastructural changes were observed under a HT-7800 transmission electron microscope (Hitachi, Tokyo, Japan).

### 4.20. Statistical Analysis

Data computation was accomplished using SPSS software 16.0 (SPSS Inc., Chicago, IL, USA). For determining the significance of differences between two groups or among multiple groups, Student’s *t*-test or one-way ANOVA was applied. Each experiment was conducted for three times at minimum. The statistical significance in differences was confirmed when *p* < 0.05.

## 5. Conclusions

In summary, we have shown that, in vivo, astrocytes may represent the earliest sign of aging in numerous types of brain cells, and senescent astrocytes could trigger neurons’ death in both direct and indirect ways. However, future investigation of cell-to-cell interactions and their mechanisms is needed. Astrocytes senescence is a brand-new field of study, and more research needs to be designed and implemented so that the autonomous and non-autonomous mechanisms of senescent astrocytes and their relationship with age-related neurodegenerative diseases can eventually be uncovered.

## Data Availability

All the applicable data have been provided in the manuscript. The authors will provide additional details if necessary.

## Supplementary Materials

The following supporting information can be downloaded at: https://www.mdpi.com/article/10.3390/molecules27185925/s1, Figure S1: The viability of astrocytes treated with different concentration of Berberine. Figure S2: The intracellular ATP level of young and aged astrocytes.

## Sample Availability

The samples used in this study are not available.
