# Supplementary material for: Senescent Phenotype of Astrocytes Leads to Activation of BV2 Microglia and N2a Neuronal Cells Death
Source: Molecules. 2022 Sep 12;27(18):5925. doi: 10.3390/molecules27185925 (PMC9506220; doi:10.3390/molecules27185925)
Supplement: Supplementary file 1 [file molecules-27-05925-s001.zip › molecules-1853165-supplementary.pdf]

molecules

MDPI

Supplementary information 1

Figure S1 The viability of astrocytes treated with different concentration of Berberine. 2

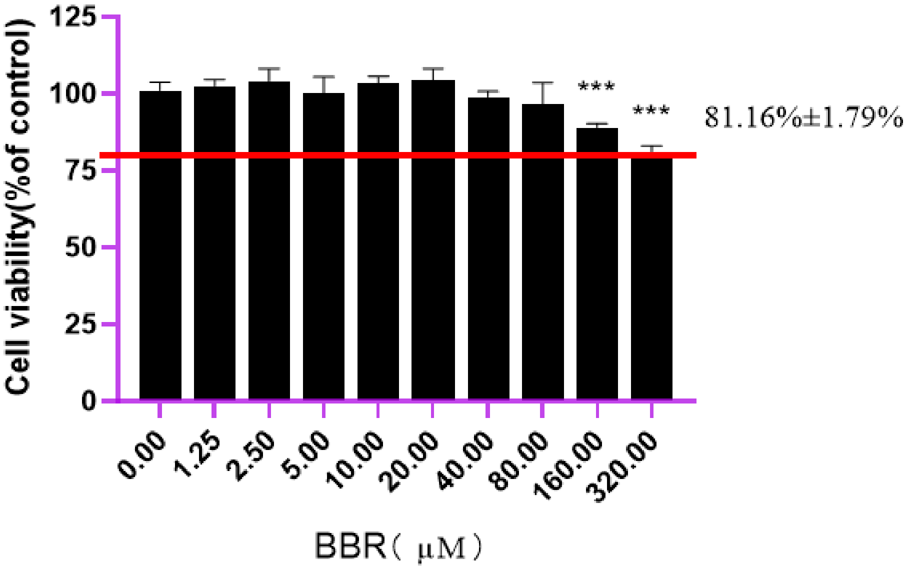

3

4
